# Ultrafast Excimer Formation and Solvent Controlled Symmetry Breaking Charge Separation in the Excitonically Coupled Subphthalocyanine Dimer

**DOI:** 10.1002/anie.202101572

**Published:** 2021-03-30

**Authors:** Palas Roy, Giovanni Bressan, Jacob Gretton, Andrew N. Cammidge, Stephen R. Meech

**Affiliations:** ^1^ School of Chemistry University of East Anglia Nowich NR4 7TJ UK; ^2^ Department of Life Sciences Imperial College London London SW7 2BX UK

**Keywords:** excimer, excited states, subphthalocyanine, symmetry breaking charge separation, ultrafast dynamics

## Abstract

Knowledge of the factors controlling excited state dynamics in excitonically coupled dimers and higher aggregates is critical for understanding natural and artificial solar energy conversion. In this work, we report ultrafast solvent polarity dependent excited state dynamics of the structurally well‐defined subphthalocyanine dimer, μ‐OSubPc_2_. Stationary electronic spectra demonstrate strong exciton coupling in μ‐OSubPc_2_. Femtosecond transient absorption measurements reveal ultrafast excimer formation from the initially excited exciton, mediated by intramolecular structural evolution. In polar solvents the excimer state decays directly through symmetry breaking charge transfer to form a charge separated state. Charge separation occurs under control of solvent orientational relaxation.

## Graphical abstract

Solvent polarity tunes the interplay of excimer formation and symmetry breaking charge separation (SB–CS) in the photoexcited subphthalocyanine dimer.

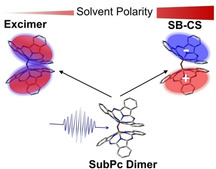

Self‐assembled chromophore aggregates play central roles in light harvesting and energy transport processes which underpin photosynthesis, solar energy conversion and molecular electronics.^[1]^ Co‐facial molecular homodimers are the fundamental unit of such aggregates and exhibit a range of phenomena including: exciton formation and decay, which is critical in efficient energy transport; relaxation to excimers, which act as trap states, disrupting exciton diffusion; symmetry breaking charge separation, a model system for the primary step in bacterial photosynthesis.^[2]^ In this work we probe ultrafast dynamics in the structurally well‐defined μ‐oxo‐subphthalocyanine dimer (μ‐OSubPc_2_), resolving sequential exciton relaxation to form an excimer, and observing its subsequent decay in polar solvents by symmetry breaking charge separation. While important intermediates in the decay dynamics of excitonically coupled dimers have been studied previously, most notably in perylene derivatives,^[3]^ the rigid and soluble μ‐OSubPc_2_ allows observation of discrete spectra for each state and their sequential kinetics in real‐time under solvent control. Thus, this study of μ‐OSubPc_2_ permits the real time characterization of relaxation through the key intermediates of photoexcited dimers; the observation of these states suggests potential applications in photovoltaic cells.

Boron SubPc has three conjugated heteroaromatic rings, in contrast to the four familiar in phthalocyanines, and consequently adopts a bowl‐like rather than planar structure. It has an intense blue‐green absorption, making it a particularly useful chromophore for solar energy harvesting.^[4]^ The oxygen bridged dimer, μ‐OSubPc_2_, has been synthesized and structurally characterized as a co‐facial non‐co‐planar dimer with approximate *C*_2*v*_ symmetry (Figure 1 a).^[5]^ The electronic spectra of μ‐OSubPc_2_ have been studied.^[6]^ The absorption shows strong exciton coupling, yielding an intense blue shifted spectrum, while the emission spectra are broad and featureless and the quantum yield is low, suggestive of excimer formation (Figure 1 b). Beyond that, the photophysics of μ‐OSubPc_2_ are uncharacterized. In the following we show that the rigid structure and good solubility of μ‐OSubPc_2_ allows us to solvent tune its photophysics and thus unambiguously resolve the dynamics of the full range of excited state processes in excitonically coupled dimers.

**Figure 1:**
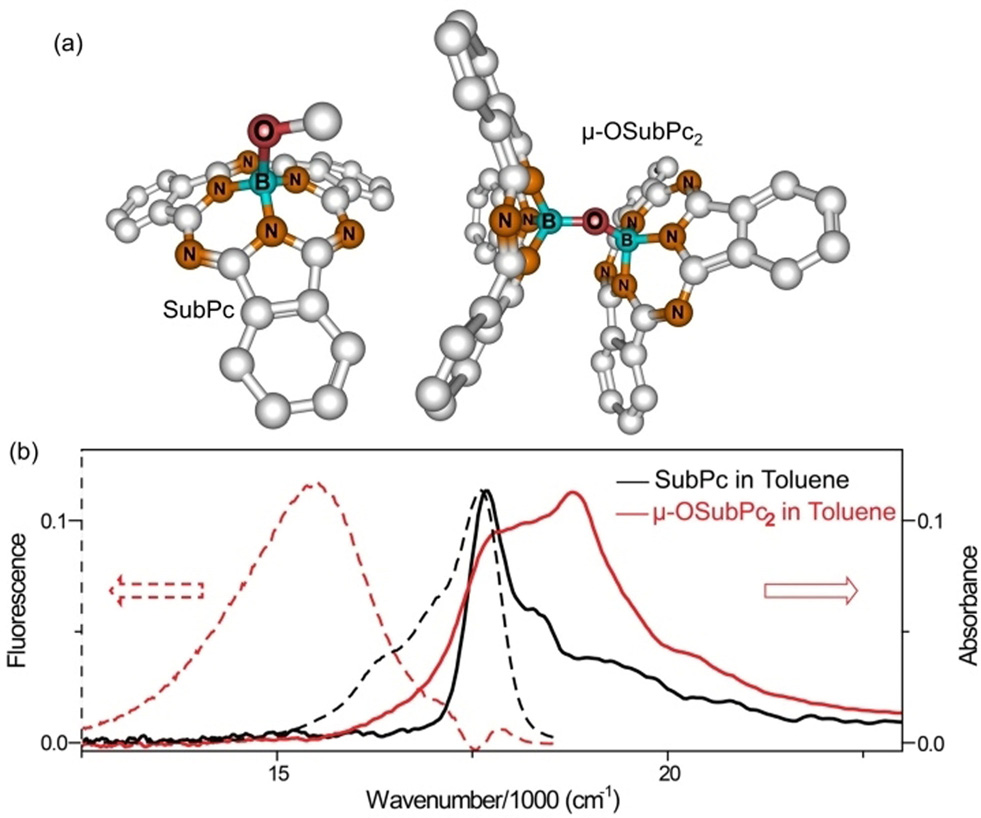
a) DFT optimized chemical structure of the SubPc monomer and μ‐OSubPc_2_ dimer. b) Absorption (solid lines) and emission (*λ*_ex_=530 nm, dash lines) spectra of monomer (black) and μ‐OSubPc_2_ dimer (red) in toluene. Spectra are shown peak normalized.

Figure 1 b shows that the absorption spectrum of the dimer has its peak absorbance blue shifted by 1110 cm^−1^ with respect to the monomer, indicating interchromophore coupling. In the calculated structure (Figure 1 a) the B–B distance is 0.265 nm, the shortest distance between π systems is 0.36 nm, and the monomer extinction coefficient is 9×10^4^ M^−1^ cm^−1^, putting μ‐OSubPc_2_ in the region where strong dipole‐dipole coupling is expected. The spectroscopy of such molecular dimers (and higher aggregates) was elucidated by Kasha, who showed that the monomer absorption is split into two exciton states in the dimer.^[7]^ For co‐facial dimers, where the transition dipole moment is in the molecular plane (H‐aggregates), the allowed (in‐phase) exciton transition is blue shifted, while out‐of‐phase transitions to the lowest energy, red‐shifted state are forbidden (in the absence of disorder). For dimers with an end‐on alignment (J aggregates) the lowest energy exciton state is allowed. In μ‐OSubPc_2_ the centres of the rings are aligned as in an H‐dimer, but the nonlinear B‐O‐B bridge imposes a pseudo *C*_2*v*_ structure (the individual SubPc units in the dimer undergo nearly barrierless rotation about the B−O bond, Supporting Information, Figure S1) yielding a non‐parallel arrangement. In this case transitions to both the lower and upper exciton states are allowed, consistent with the simultaneous observation of a blue shifted maximum and absorption to the red of the monomer 0–0 transition in μ‐OSubPc_2_ (Figure 1 b).^[7]^

In contrast to the structured asymmetric absorption, the emission is broad and featureless, and exhibits a large Stokes loss. This spectrum is consistent with emission from an intramolecular excimer state, as detailed further below. Note that emission on the blue edge (near 17 610 cm^−1^) is ill‐defined in Figure 1 b because a trace‐monomer impurity emission has been subtracted. This contribution is superficially similar to emission from a Frenkel exciton state (as has been seen in perylene dimers^[3c]^) but detailed analysis shows it to be a monomer contribution (Supplementary information Figure S2).

Transient absorption (TA) spectra (Figure 2) of μ‐OSubPc_2_ excited at 546 nm were measured with ca 100 fs time resolution between 400 and 1300 nm, in five solvents of widely varying polarity and solvation time, specifically: toluene (TL), methyltetrahydrofuran (MTHF), acetonitrile (ACN), *N*,*N*‐dimethylformamide (DMF) and 1:1 TL/ACN mixture. Experimental details are described elsewhere and in the Supporting Information.^[8]^ The TA of the SubPc monomer has been published elsewhere^[9]^ and is shown in the Supporting Information Figure S3 for reference. It shows an intense bleach at 17 690 cm^−1^ and weaker induced absorption to the red (12 000–17 000 cm^−1^) and blue (peaking at 21 200 cm^−1^). The former decays in ca 2 ns as the bleach partially fills, while the latter does not evolve suggesting both singlet‐singlet and triplet‐triplet absorption contribute to that transient absorption, consistent with literature observations.^[10]^ There is no TA observed for the monomer below 11 000 cm^−1^.

**Figure 2:**
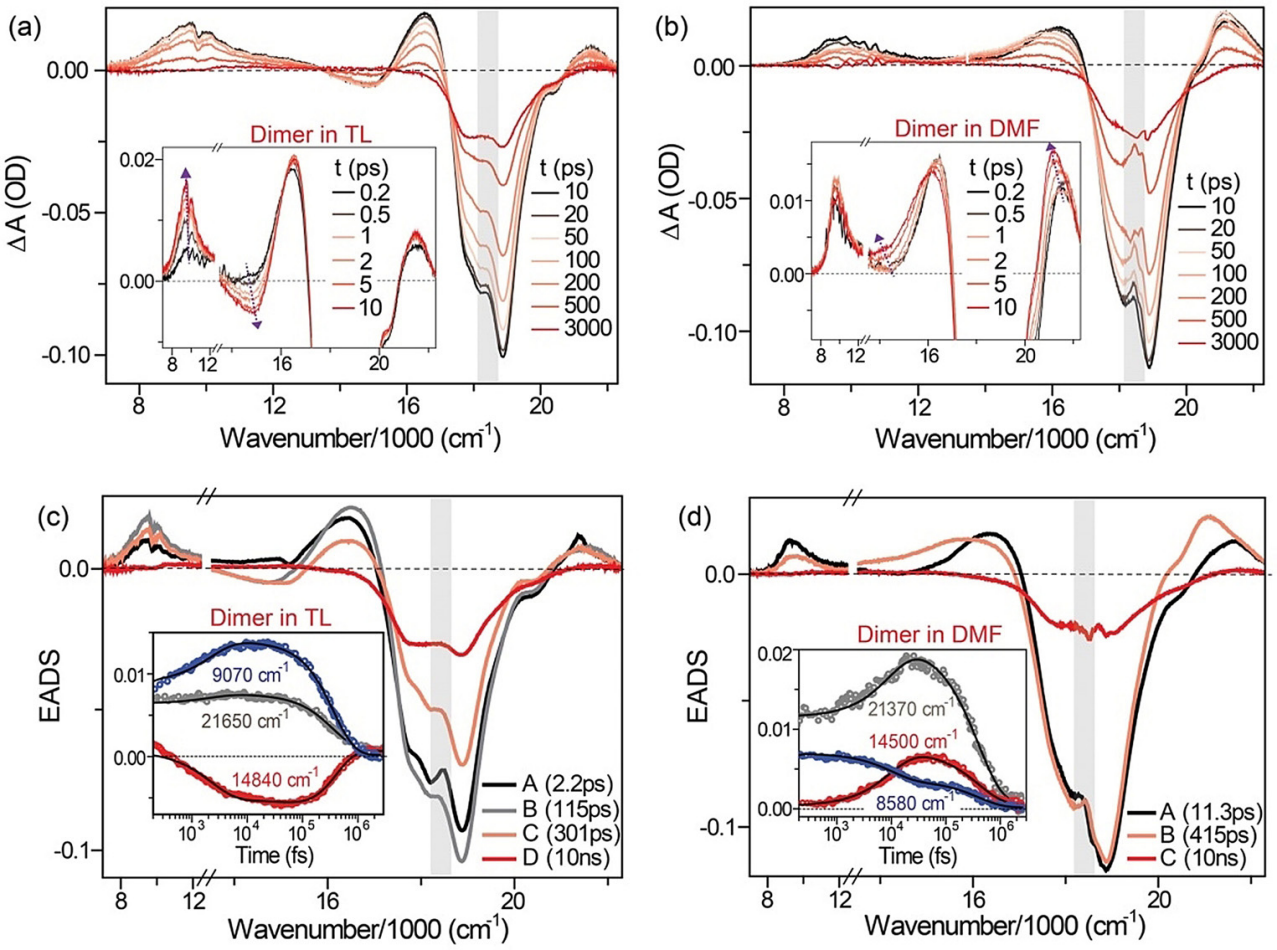
a,b) Transient absorption spectra of μ‐OSubPc_2_ dimer in toluene (TL) and DMF respectively at different timescales, with insets showing early time data. Excitation was at 546 nm and the dimer concentration was 12 μM. c,d) EADS recovered from global analysis for the TA datasets in TL and DMF respectively. Inset shows data and global analysis fits at different probe wavenumbers. Note that data near 18600 cm^−1^ are perturbed by scattered light (shown by grey area).

Figure 2 shows the TA for μ‐OSubPc_2_ in TL and DMF (data for MTHF, ACN and ACN/toluene mixtures are shown in the Supporting Information, Figure S4). In toluene (Figure 2 a) neither the bleach nor the TA, which appear promptly at 16 500 and 21 500 cm^−1^ respectively, evolve significantly in the first 10 ps. However, a negative feature appears on the picosecond timescale at 14 900 cm^−1^; based on the absence of ground state absorption and the location of the excimer fluorescence this is consistent with stimulated emission from the excimer (Figure 1 b); the shift compared to Figure 1 b arises from the overlapping TA (Figure S7). At the same time a transient grows in the near IR (NIR) at 9400 cm^−1^. A similar NIR transient was observed in the perylene excimer and was assigned as an excimer to charge transfer state transition.[^3d^, ^3e^] After about 10 ps all transient states relax toward the baseline, although the ground state does not fully recover, and at 3 ns there is a weak residual absorption at 21 600 cm^−1^, which is assigned to population of a long lived, possibly triplet state (the triplet TA is reported at 21 700 cm^−1^ in SubPc films).^[10]^

Figure 2 b reveals very different behaviour for μ‐OSubPc_2_ in DMF. In this case stimulated emission associated with excimer formation is not observed (consistent with the very weak broad steady state emission in this solvent, supplementary information Figure S2d,e). Instead the spectral evolution shows a picosecond rise in the transient absorption at 21 200 cm^−1^ and a broadening in the 12 000–17 000 cm^−1^ transient, accompanied by a low amplitude rise in the NIR band, which probably reflects the same underlying broadening (see global analysis below). We note that in μ‐OSubPc_2_ the NIR band does not shift with solvent polarity suggesting that in the μ‐OSubPc_2_ dimer it is not a transition to a charge separated state.

In the weakly polar MTHF solvent intermediate behaviour is observed, with both stimulated emission due to excimer formation along with a slower rise and broadening in the transients in both visible and NIR (See the Supporting Information, Figure S4b). For polar ACN the spectral evolution is the same as for DMF, but significantly faster (See the Supporting Information, Figure S4c).

Quantitative analysis of the TA dynamics was performed by global analysis,^[11]^ where time resolved data were analyzed with a sequential model, for which only one or two intermediate states (for polar and nonpolar solvents, respectively) plus a final state were required to obtain an accurate fit. The resulting evolution associated difference spectra (EADS) for toluene and DMF are shown in Figures 2 c and d, respectively, while those for MTHF and acetonitrile are given in the Supporting Information, Figure S5, and all kinetic data are tabulated in the Supporting Information (Table S1). To show the quality of fit, the single wavelength data for key transients are plotted in the inset of Figure 2 c and d, including the fitted function from the global analysis.

In nonpolar toluene the first two components are required to describe the formation of the excimer stimulated emission (at 14 840 cm^−1^, Figure 2), which initially appears in 2.2 ps. Significantly, the NIR absorption (9070 cm^−1^) appears promptly on population of the dimer excited state, although it grows in amplitude as the stimulated emission develops. Thus, this NIR TA is evidently a dimer band rather than one specifically associated with the excimer, although the observed time dependence shows that its transition moment depends on the state (exciton or excimer) of the dimer. The second slower component in excimer formation is essential for a good fit (see the Supporting Information, Figure S6a) and appears in evolution of both the stimulated emission and the 16 500 cm^−1^ transient absorption. We assign this slower component to reorganization in the excimer structure during its lifetime. The rigid μ‐OSubPc_2_ structure leaves little room for major structural reorganization. One possibility is evolution in the angle formed by the two rings, which would require solvent displacement. Alternatively, it is possible that in place of the free rotation about the O−B bonds observed in the ground state (Figure S2), the excimer has a favoured low energy orientation of the two rings, which is adopted in ca 100 ps. After this excimer reorganization the only further evolution observed in toluene is a 301 ps uniform decay in population, which yields a final long lived state with a spectrum similar to that of the SubPc triplet.^[10]^

In contrast, global analysis of μ‐OSubPc_2_ in polar DMF has only a single intermediate which forms in 10 ps, (Figure 2 d). This is characterized by the absence of a stimulated emission contribution from the excimer, and a strong rising component at 21 370 cm^−1^, accompanied by a broadening of the transient absorption on the low wavenumber side of the bleach, and a decrease in amplitude of the NIR band (which however persists). We assign these kinetics to polar solvent induced symmetry breaking charge separation in the μ‐OSubPc_2_. The growth in absorption around 21 370 cm^−1^ and 14 500 cm^−1^, which extends across much of the visible range, is consistent with the spectrum of the SubPc cation observed in a mixed SubPc:C_60_ film, where electron transfer occurs;^[10]^ in that film characteristic TAs of the transient cation at 19 200 and 14 000 cm^−1^ are reported, while the chemically oxidized SubPc shows a strong absorption around 15 000 cm^−1^. The spectrum of reduced SubPc was observed in a photoelectron transfer and pulse radiolysis study, revealing a strong feature near 21 000 cm^−1^, as seen here in the TA.^[12]^ Thus, comparison with the available literature is consistent with charge separation in the μ‐OSubPc_2_ dimer.

The TA data in the intermediate polarity solvent MTHF again requires two intermediate components for a successful fit (see Supporting Information, Figure S6b), but the spectroscopic behaviour is quite distinct from that in toluene. The first 1.3 ps relaxation reflects formation of the excimer stimulated emission, but the subsequent 7 ps step is associated with a small rise in the transient absorption near 21 500 cm^−1^ and a broadening in the transient spectrum around 15 000 cm^−1^ (see also the Supporting Information, Figures S4, S5). This second step is therefore associated with formation of the charge separated (CS) state from the excimer or, since transient spectra of both persist throughout the subsequent 282 ps decay, it is more likely that on the 7 ps timescale an equilibrium is established between excimer and CS state.

The kinetics in acetonitrile provide further support for symmetry breaking charge separation in polar media. The spectral evolution is essentially identical to that in DMF, but the kinetics are on a faster time scale. We assign this difference to solvent control of symmetry breaking charge separation. The mean solvation time of acetonitrile (0.26 ps) is faster than that of DMF (0.9 ps) in line with this assignment.^[13]^ However, these times are clearly both faster than observed for formation of the CS state. These solvation times are dominated by a fast inertial (librational) solvation component. A recent measurement and simulation of charge transfer state formation in bianthryl suggests that the solvent librational component is not effective in stabilizing charge separation.^[14]^ In bianthryl the slower diffusive orientational solvent modes were shown to be critical in the stabilization. Extending that argument to μ‐OSubPc_2_, we have for ACN a 0.6 ps diffusive response, which is close to the 1.2 ps observed. For DMF the slower solvation dynamics are more complex, with components of 2 and 30 ps contributing, both notably longer than for ACN.^[13]^ Thus, the observed kinetics are consistent with the symmetry breaking decay of the excimer to form the CS state being under the control of diffusive polar solvent reorientation. The asymmetry required is introduced by a fluctuation in the polar solvent environment surrounding the symmetric excimer. The same mechanism has been shown to operate for charge separation in quadrupolar chromophores.^[15]^ These conclusions are summarized in Figure 3.

**Figure 3:**
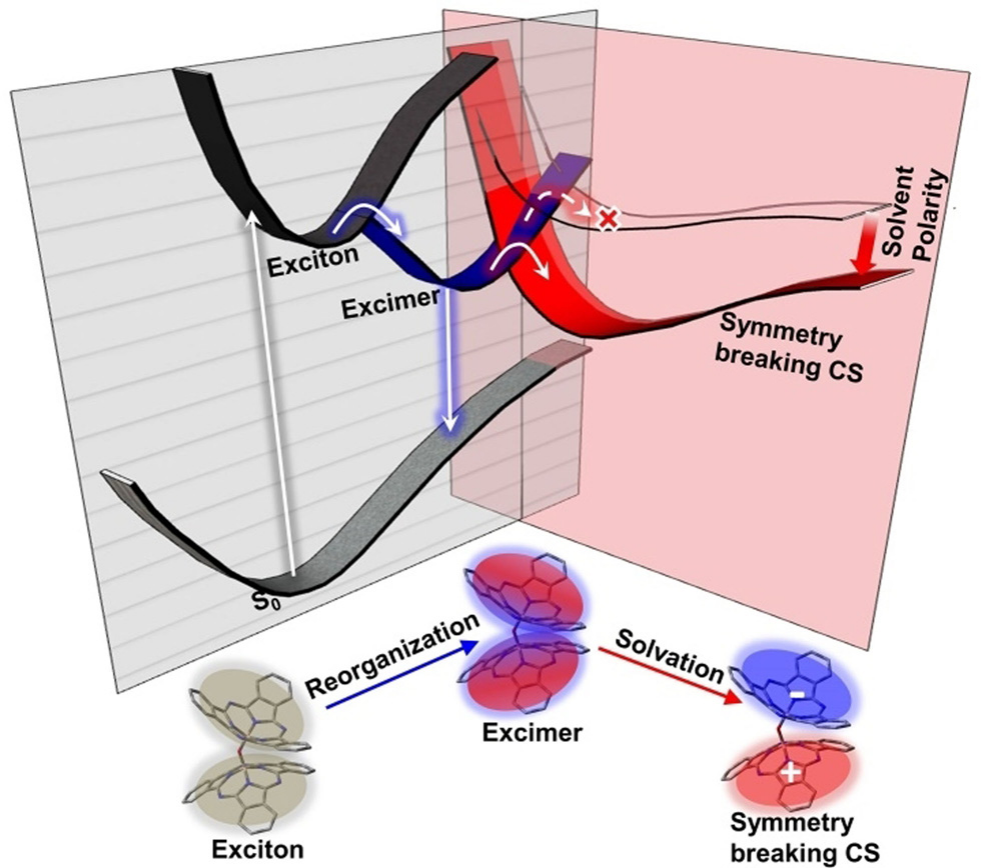
Schematic representation of μ‐OSubPc_2_ photophysics. Direct excitation is to a Frenkel exciton state, where excitation is shared over the dimer. This is followed by sequential formation of the μ‐OSubPc_2_ excimer and its decay by symmetry breaking charge separation to form a charge separated state, which is accessible only in polar solvent. The first step thus involves an evolution in the wavefunction of the Frenkel exciton state to favor charge resonance forms in the excimer. This involves evolution on intramolecular coordinates. The excimer subsequently decays along a solvation coordinate in polar solvents, with the initial step arising from an asymmetric fluctuation in the solvent environment.

In summary, the transient excited state dynamics of the strongly excitonically coupled, structurally well‐defined μ‐OSubPc_2_ have been recorded in a range of solvents. The formation of an excimer state from the initially excited dimer was observed to occur on a picosecond timescale. In solvents of moderate to high polarity, solvent orientational fluctuations introduce an asymmetry which promotes ultrafast decay of the excimer to a CS state. The observation of these sequential steps in a single structurally well‐defined dimer suggests μ‐OSubPc_2_ is a good candidate for modelling excited state dynamics in excitonically coupled dimers. Further, we note that the μ‐OSubPc_2_ itself has a potentially important role to play in photovoltaic devices. Its intense blue shifted broad absorption is helpful for harvesting solar energy,^[16]^ and its ability to yield intramolecular charge separation in asymmetric environments is useful in photovoltaic applications, because CS state formation may reduce the binding energy of charge carriers, which can thus be more easily dissociated and extracted in photovoltaic devices.^[17]^

## Conflict of interest

The authors declare no conflict of interest.

## Supporting information

As a service to our authors and readers, this journal provides supporting information supplied by the authors. Such materials are peer reviewed and may be re‐organized for online delivery, but are not copy‐edited or typeset. Technical support issues arising from supporting information (other than missing files) should be addressed to the authors.

Supplementary
