# Supplementary material for: Ultrafast Excimer Formation and Solvent Controlled Symmetry Breaking Charge Separation in the Excitonically Coupled Subphthalocyanine Dimer
Source: Angew Chem Int Ed Engl. 2021 Mar 30;60(19):10568–72. doi: 10.1002/anie.202101572 (PMC8251754; doi:10.1002/anie.202101572)
Supplement: Supplementary file 1 — Supplementary [file ANIE-60-10568-s001.pdf]

Angewandte Chemie
Eine Zeitschrift der Gesellschaft Deutscher Chemiker

Supporting Information

Ultrafast Excimer Formation and Solvent Controlled Symmetry Breaking Charge Separation in the Excitonically Coupled Subphthalocyanine Dimer

*Palas Roy, Giovanni Bressan, Jacob Gretton, Andrew N. Cammidge, and Stephen R. Meech**

anie_202101572_sm_miscellaneous_information.pdf

Supporting Information

Experimental Details

Figure S1 Free Ground State Rotation at Room Temperature

Figure S2 Characterization and Subtraction of Monomer Impurity Emission

Figure S3 Monomer Transient Absorption

Figure S4 Additional TA data for ACN, MTHF and Toluene:ACN Mixture

Figure S5 Global Analysis for Figure S4

Figure S6 Analysis for Toluene and MTHF Showing Requirement for Two Kinetic Intermediate

Figure S7 Comparison of steady state emission and TA

Figure S8 Absence of concentration effects on TA

Table S1 Results from Global Analysis Fitting

Synthesis and Characterization

Experimental Details

Femtosecond transient absorption (TA) measurements. TA is two pulse technique where a femtosecond actinic pulse (as pump) excites the molecule of interest and a broadband white light continuum (as probe) monitors the excited state. The detailed description of the transient absorption set up has been mentioned elsewhere.[1] Briefly, Ti:sapphire regenerative amplifier (Spectra Physics Spitfire ACE) is used to amplify the fundamental beam from the Spectra Physics Spitfire Mai Tai Laser oscillator. The amplified output pulse (duration of 120 fs at 800 nm at a repetition rate of 1 kHz and energy of 5 mJ per pulse) then drives two commercial optical parametric amplifiers (OPA, Light Conversion TOPAS Prime). One OPA generates the 546 nm actinic pump pulse with duration of 80fs which is passed through a mechanical chopper (at 500Hz), a computer controlled translational stage (to provide delay between pump and probe pulses) and a polarizer (to set polarization at magic angle). The second OPA is tuned to generate 1250nm pulse which was attenuated (10uJ) and then focused onto a 3 mm thick sapphire window to generate broadband white light continuum (WLC) that is used for collecting spectra in the window 550-1400nm. To capture spectra in the blue side (400-740nm), the fundamental beam (instead of sending it to the second OPA) was directly passed through the sapphire plate to generate WLC. The WLC before the sample stage was split by a 50/50 beam splitter- one part (as Probe) was passed through a 2 mm thick static cuvette with quartz glass window and the second part (as Reference) was used in reference detection channel to correct for intensity fluctuations in the probe spectrum. Both the actinic pump and probe pulses were focused and overlapped spatially and temporally inside the cuvette. The focal spot size for pump and probe pulses were kept 270 µm and 50 µm respectively. Pump pulse energy was attenuated to 0.25 mW at the sample cell. Absorption spectra of the sample was taken before and after measurements to ensure photostability of the sample. Absorbance was kept below 0.5 OD in 2 mm cuvette for all the TA measurements. After the sample stage, probe was aligned collinearly with reference beam on top of each other using a mechanical slit. Both the beams are then dispersed using a prism based spectrograph and detected by two separately synchronised 16 bit A/D CCD detectors from Entwicklungsbüro Stresing (201*1024 pixels).

The detection of pump-on/pump-off for probe and reference spectra was possible by placing a chopper at 500Hz in the actinic pump beam path. The referenced difference spectrum was calculated for each pulse pair using the following equation:

$$\Delta A = -log\left(\frac{Probe\ (Pump\ on)\ \mathrm{X}\ Refernce\ (Pump\ off)}{Reference\ (Pump\ on)\ \mathrm{X}\ Probe\ (Pump\ off)}\right)$$

The detector was calibrated using Hg-lamp (HG-1-spectrum-with-USB2000-XR1-2 Ocean Optics). Solvent response using 546 nm excitation pump and WLC_{Sig} probe provided the instrument response function, IRF measured to be about 100 fs. The data presented was averaged over 4 cycles and each time trace was accumulated for 1sec. Accurate TA spectra were extracted by removing static scatter of pump followed by correcting the group velocity dispersion (chirp) of the white light. Scattered pump radiation at 546 nm (18315 cm^{-1}) perturbed some TA so data in this region are omitted. Glotaran software[2] with a sequential model was used to fit the data with 3 or 4 exponential kinetic and spectral parameters.

Time-resolved and steady-state fluorescence: Steady-state Fluorescence and excitation spectra of monomer and dimer were recorded in Edinburgh Instruments FS5 Spectrofluorometer equipped with continuous (150 W) xenon lamp. Time-resolved fluorospectroscopy were conducted using the spectrometer based on a time correlated single photon counting (TCSPC) technique under the excitation of a 485 nm picosecond laser. The lifetimes were determined via the equation: $\tau=\int I(t)dt/I_0$,

where I(t) is the emission intensity at time t and I_0 is the peak intensity. 1cm path length cuvette was used for all the measurements.

DFT Calculations: Geometry optimisations and the energy calculations were carried out with the GAUSSIAN 16 program within the DFT approach, using the RB3LYP/tzvp method. In order to study the barriers to internal rotation, the geometries were optimized for different fixed internal rotation angles (NBOB dihedral angles).[3]

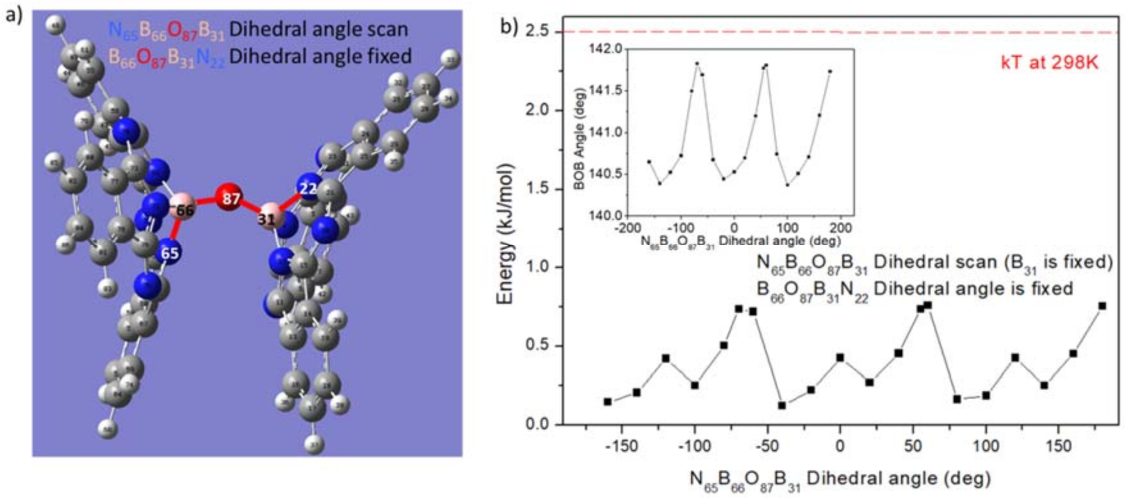

Figure S1. DFT calculated geometry optimised energy of μ-OSubPc$_2$ was obtained at different fixed NBOBN geometries, determined by the two NBOB dihedrals (as shown in (a)). The potential-energy profiles (b) for rotation of one ring with respect to the other is calculated with one dihedral fixed and the other rotated about the BO axis in 10-20° steps. Interestingly the potential is not exactly three fold symmetric, which shows that other coordinates are involved in the rotation. An important one is the BOB angle (inset in (b)). There were also suggestions of minor distortions to the SubPc framework, but these were not investigated. The central result is the low barrier to rotation about the BO bonds.

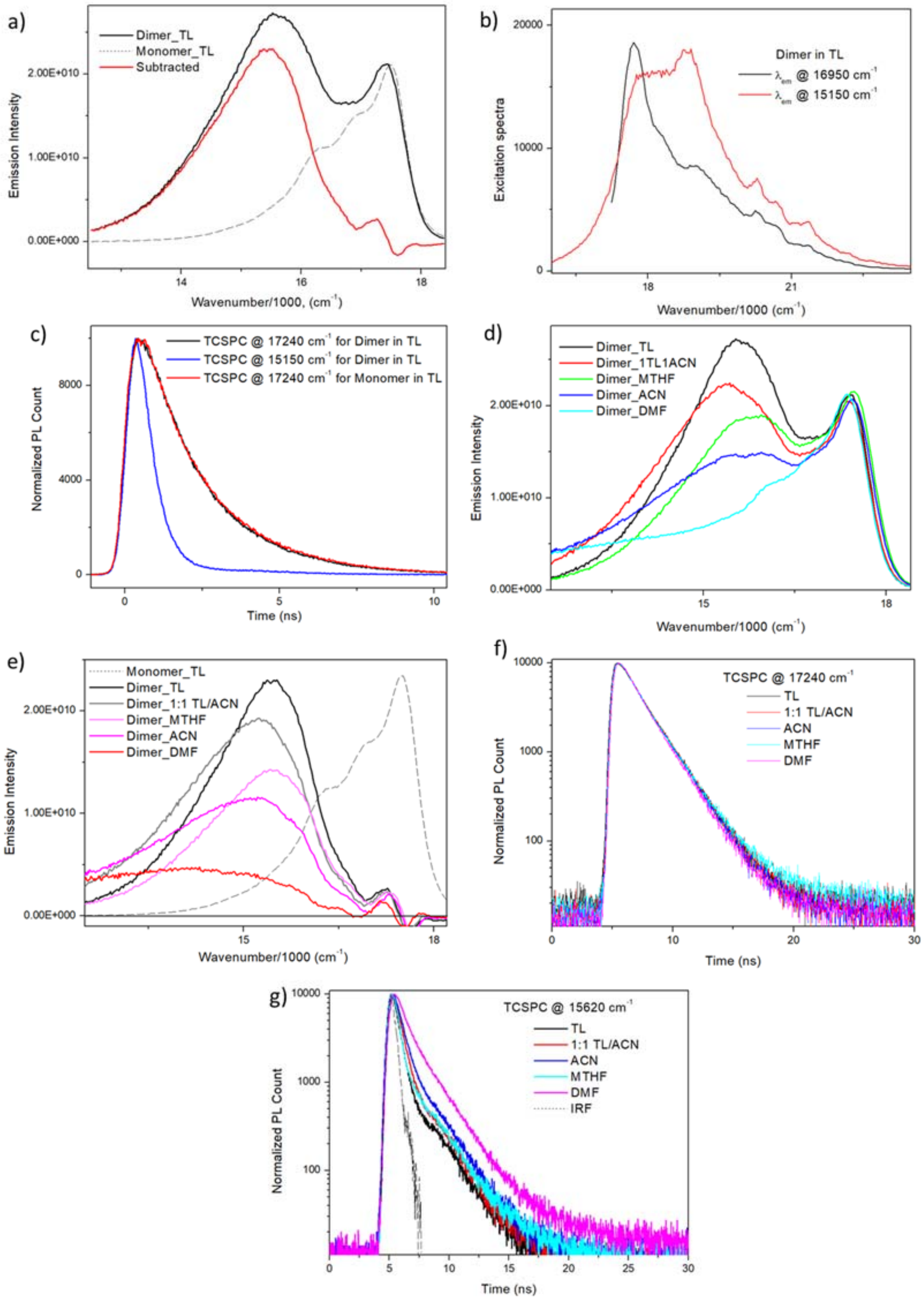

Figure S2. a) The emission spectra of μ-OSubPc$_2$ show two bands (black spectra showing peaks at 15150 and 17610 cm^{-1}). One plausible assignment is that this is a result of Frenkel exciton emission (structured) in competition with decay to the broad featureless red-shifted excimer emission. However, this assignment is not supported by subsequent observation. (a) Shows that the emission can be separated into a sum of an excimer like and monomer like emission. Evidence against the blue structured emission being associated with the directly excited exciton is that no such features appear in the TA, although the monomer like contribution to the emission appears significant (Compare Figure 2 main text with S3, for the monomer, below). These results are better understood as emission from a minor monomer impurity of high fluorescence yield. b) Excitation spectra uncover multiple ground states, which contribute two distinct emissions. Excitation spectra of the

structured blue shifted part of the emission in S2a (black spectra) matches the SubPc monomer absorption, while excitation spectra of the excimer emission (red spectra) matches the measured dimer absorption. (compare with Figure 1) . The existence of multiple ground states was confirmed by the observation that the relative contribution of each component was a function of excitation wavelength (data not shown). These data are again wholly consistent with a minor contribution from a monomeric impurity. c) This interpretation was confirmed by time correlated single photon counting (TCSPC) which showed the broad red-shifted emission decay (blue trace) matched the long decay (100s of ps) seen in TA of the dimer, while the structured blue shifted emission decay (black) matched the lifetime (~2 ns) of the monomer (red) in the same solvent. The identical lifetime supports impurity emission. (d) The steady state emission spectra in a range of solvents prior to subtraction are shown (normalized for the absorbance at the excitation wavelength, so intensities can be compared). These show that the blue shifted emission is invariant with solvent while the excimer like emission is strongly solvent polarity dependent. This observation is inconsistent with the observed kinetics for exciton to excimer decay, but wholly consistent with emission from a monomer like impurity. The spectra after subtraction of the monomer contribution are shown in (e) revealing the polar solvent induced red shift and broadening. (f) Shows the nanosecond TCSPC emission of the dimer in all solvents, where again a solvent independent nanosecond emission dominates at the wavelength of the blue shifted emission. (g) Recording decay in the excimer emission region, the expected shorter lifetime is recovered. Again these results are inconsistent with the two bands being coupled in any kinetic scheme, but match the picture of a monomeric impurity. Thus, this feature is subtracted in steady state emission.

Utilizing the known quantum yield of monomer and excimer we estimate the monomer impurity to be present at a level of 2%. For this calculation we assume population of each state N_i is given by the measured intensity divided by fluorescence quantum yield, so the ratio is

$$\frac{N_m}{N_e} = \frac{I_m}{\Phi_m}\frac{\Phi_e}{I_e}$$

And the yields were taken from ref[4] and the intensities from the areas of the two spectra.

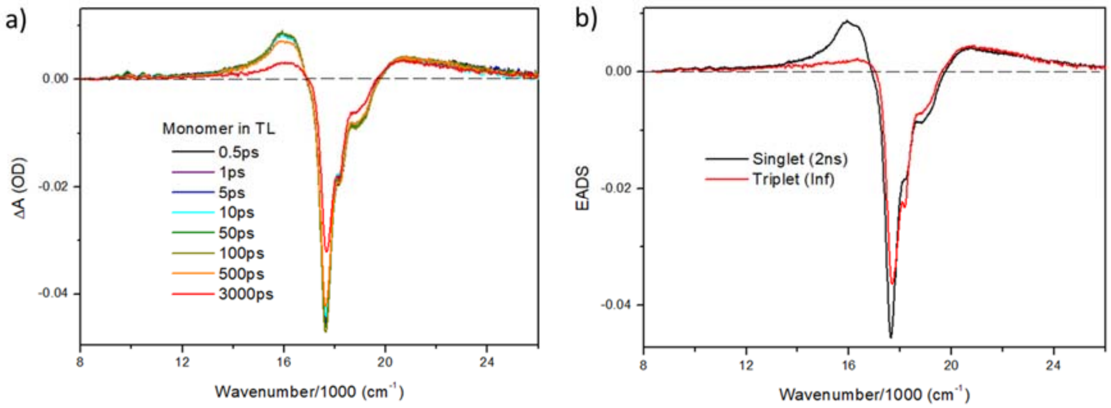

Figure S3. TA of monomer in TL was performed with pump excitation at 546 nm. a) Spectral traces at different pump-probe delays are shown. b) Corresponding Global analysis components are presented. Note the residual absorbance after 3 ns can be assigned to triplet state formation.[5]

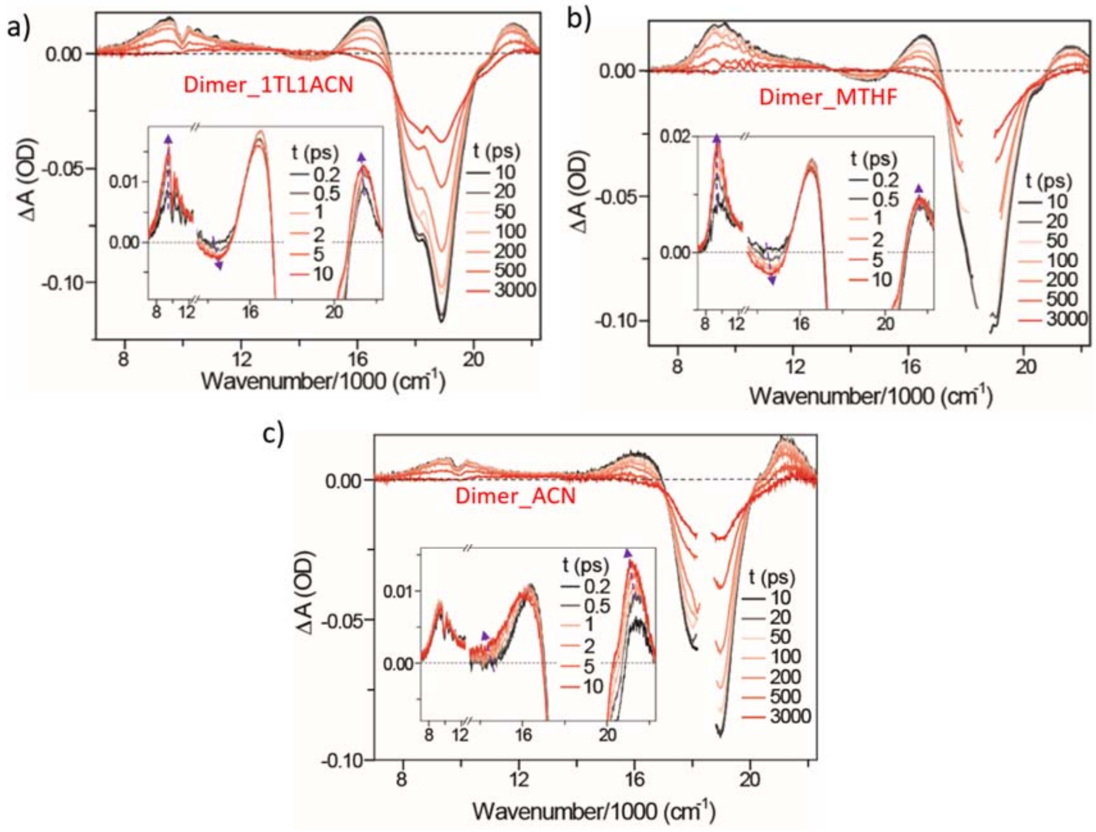

Figure S4. TA spectra with pump excitation at 546 nm for μ-OSubPc$_2$ in a) 1:1 TL and ACN mixture; b) MTHF and c) ACN are shown. The insets show initial time spectral evolutions. Gaps near 18000 cm^{-1} arise due to interference of scattered excitation light.

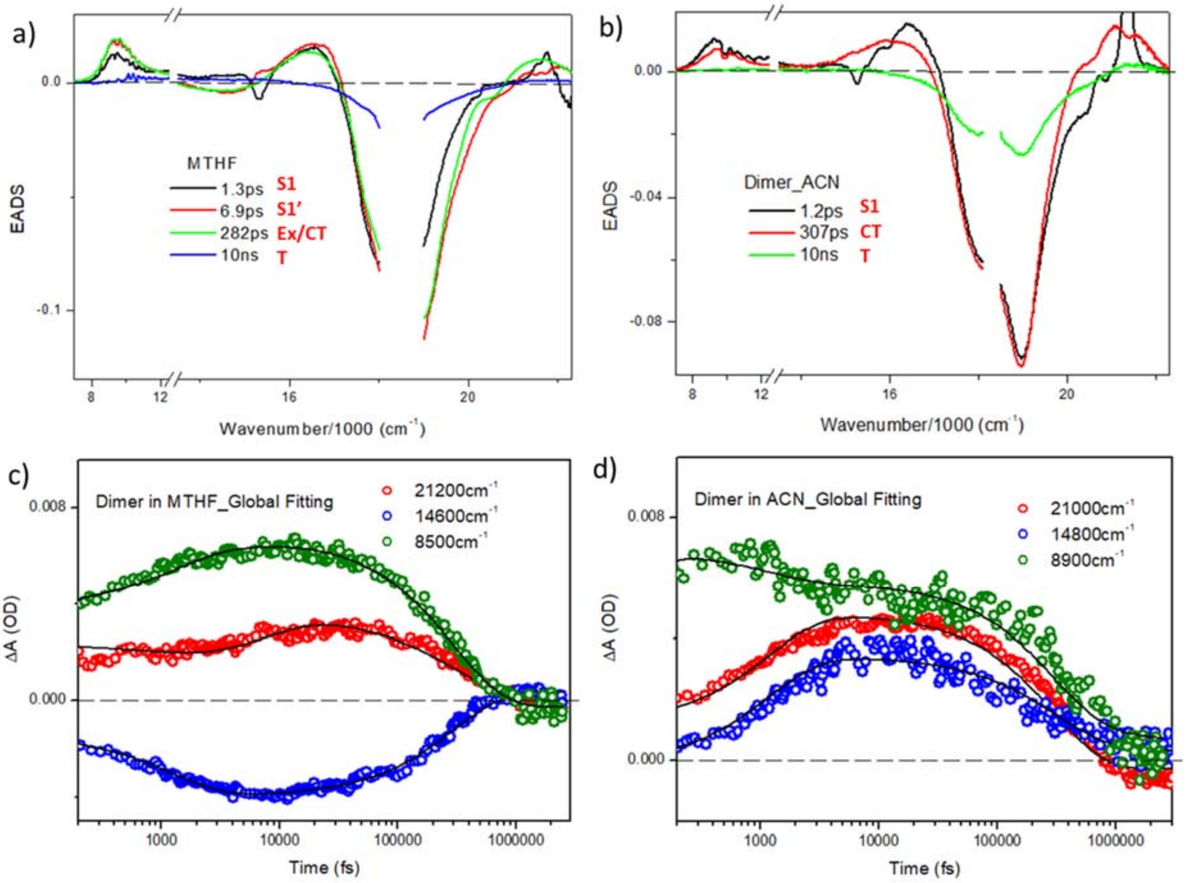

Figure S5. a, b) Global analysis components and c, d) global kinetic fittings of TA datasets for μ-$OSubPc_2$ in MTHF and ACN respectively. The TA raw data are shown in Figure S4. Gaps near 18000 cm^{-1} arise due to interference of scattered excitation light. The data for MTHF are significant as the formation of the excimer is followed by slow formation of the CT state at 21200 cm^{-1}.

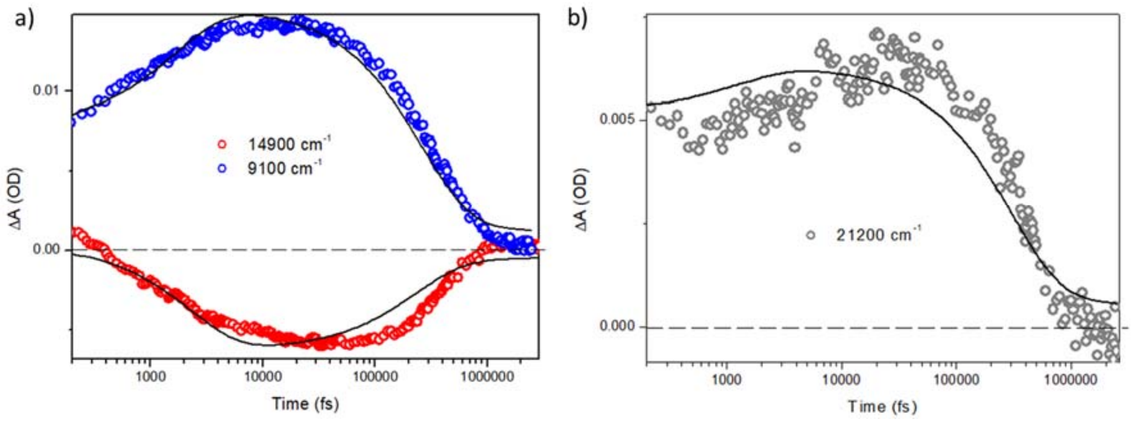

Figure S6. Global kinetic fittings assuming only a single intermediate for TA datasets for the μ-$OSubPc_2$ dimer in a) TL and b) MTHF. Data show that the fits (black line) are not good in both the solvents, requiring an additional component.

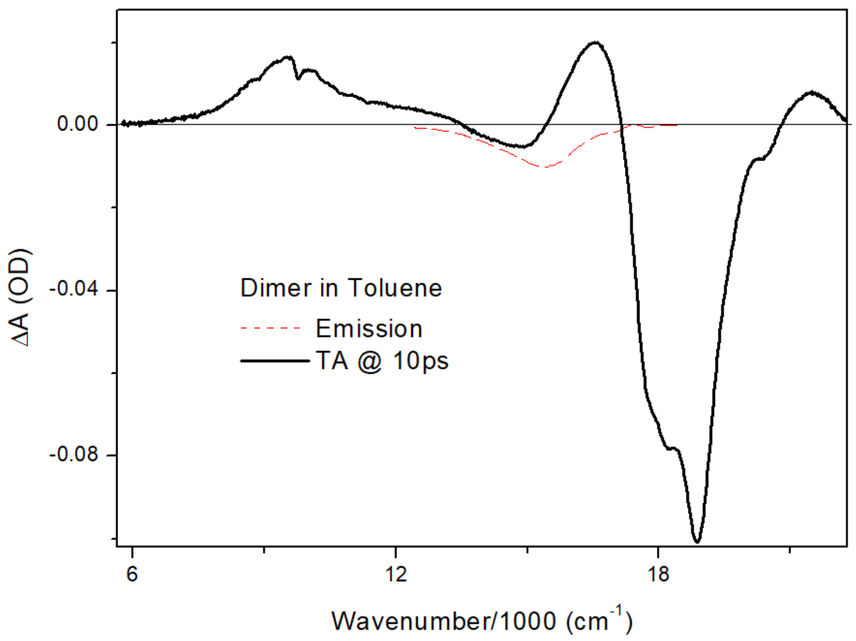

Figure S7 We plot the steady state excimer emission spectrum for the dimer alongside the TA to show the origin of the shift in the observed SE arises from the superimposed TA.

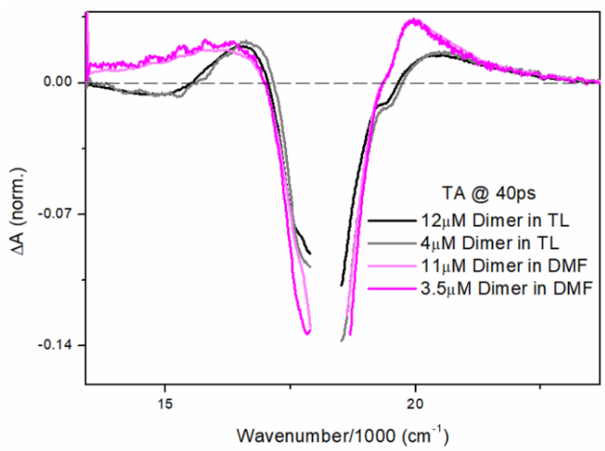

Figure S8 To eliminate the possibility of intermolecular interactions the TA data were recorded at normal and three times diluted, with identical results. In the figure the TA data are normalized for comparison.

Table S1 Lifetimes from global analysis assuming sequential evolution from initially excited Frenkel exciton state to excimer and, in polar solvents, its ultrafast decay to a CT state. The Excimer intermediate is not resolved in the most polar solvents. The final state is repopulation of the original ground state plus population in a triplet state.

μ-OSubPc$_2 \xrightarrow{h\nu}$ FE $\xrightarrow{\tau_1}$ $\xrightarrow{\tau_2}$ Excimer. $\xrightarrow{\tau_3}$.final state

μ-OSubPc$_2 \xrightarrow{h\nu}$ FE $\xrightarrow{\tau_1}$ Excimer $\xrightarrow{\tau_2}$ CT . $\xrightarrow{\tau_3}$.final state

Solvent	τ_1/ps	τ_2/ps	τ_3/ps
Toluene	2.2	115.0	301
MTHF	1.3	6.9	282
Acetonitrile		1.2	307
DMF		11.3	415

Synthesis and Characterization

General methods

Solvents were purchased from commercial sources and used without further purification unless stated. Starting materials and reagents were purchased from Merck Ltd. Phthalonitrile was recrystallised from toluene before use. Column chromatography was carried out on silica gel 60A 40-63 micron (Material Harvest Ltd). Biobeads SX-3 were purchased from Bio-RAD Labs Ltd. ^{1}H spectra were recorded at 500 MHz using a Bruker Ascend^{TM} 500 spectrometer and the residual solvent peaks were used as references. MALDI-TOF mass spectra were obtained using a Shimadzu Biotech Axima instrument.

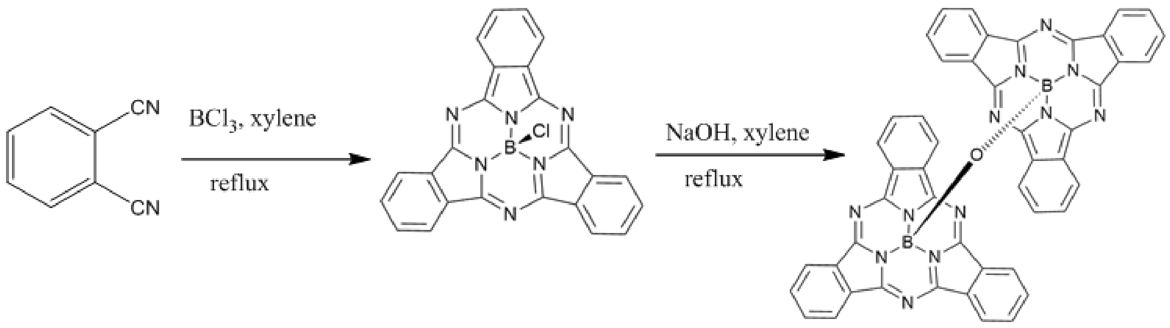

Synthesis of SubPcB-Cl[6]

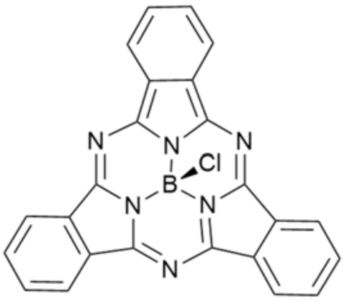

In a modified version of the reported procedure,[4] phthalonitrile (2,1 g, 16.4 mmol) was dissolved in *p*-xylene (60 mL) under N_2. To this solution 1.0M BCl_3 in *p*-xylene (11.0 mL, 11.0 mmol) was added dropwise. After complete addition, the solution was heated to reflux for 3 h, yielding an intensely purple solution. The solvent was removed *in vacuo* overnight, and the dry crude residue placed in a Soxhlet extractor. The crude material was washed with MeOH until near colourless washings were observed. The residue was subsequently extracted with chloroform for 5h, yielding a dark purple solution. The solvent was removed *in vacuo* to give the title compound as a gold/purple solid (0.89 g, 38%) which was used directly in the next step.

^{1}H NMR (500 MHz, $CDCl_3$) δ 8.89-8.92 (m, 6H), 7.94-97.98 (m, 6H); MS (MALDI-TOF): m/z = 430.62 [M, $^{35}Cl]^{+}$; UV-Vis (CH_2Cl_2): λ (nm) = 566, 305.

Synthesis of μ-Oxo-(BSubPc)$_2$ [4, 7]

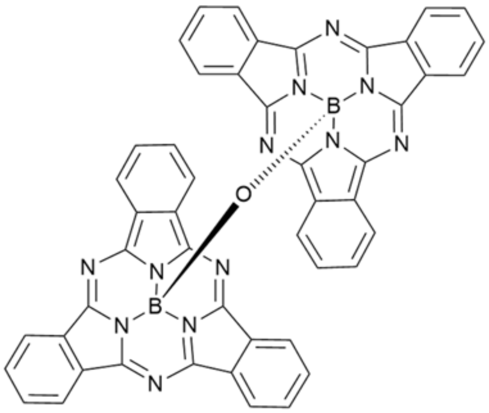

Following a modified version of the reported procedure,[7] μ-OSubPc$_2$ was obtained by heating SubPcB-Cl (20 mg, 0.046 mmol) in refluxing p-xylene (30 mL) in the presence of dibenzo-18-crown-6 (37.7 mg, 2.25 eq) and NaOH (11.1 mg, 6 eq) for 24 h. The reaction was cooled to room temperature and filtered, washed with water (2 x 50 mL) and brine (2 x 50 mL), then purified by silica gel chromatography (EtOAc : Toluene : DCM, 1 : 3 : 3). The product was recovered as a blue fraction, and recrystallised from acetone/hexane to give analytically pure (^{1}H NMR) μ-OSubPc$_2$ (5 mg, 27%). Immediately prior to measurements, and with exclusion of light for all operations, small samples were passed through size-exclusion (Biobeads SX-3) and silica gel chromatography columns (distilled toluene eluent) and the solvent removed on a rotary evaporator.

^{1}H NMR (500 MHz, Acetone-d6) δ 8.56-8.60 (m, 12H), 7.86-7.89 (m, 12H).); MS (MALDI-TOF): m/z = 809.25 [M+H]$^+$.

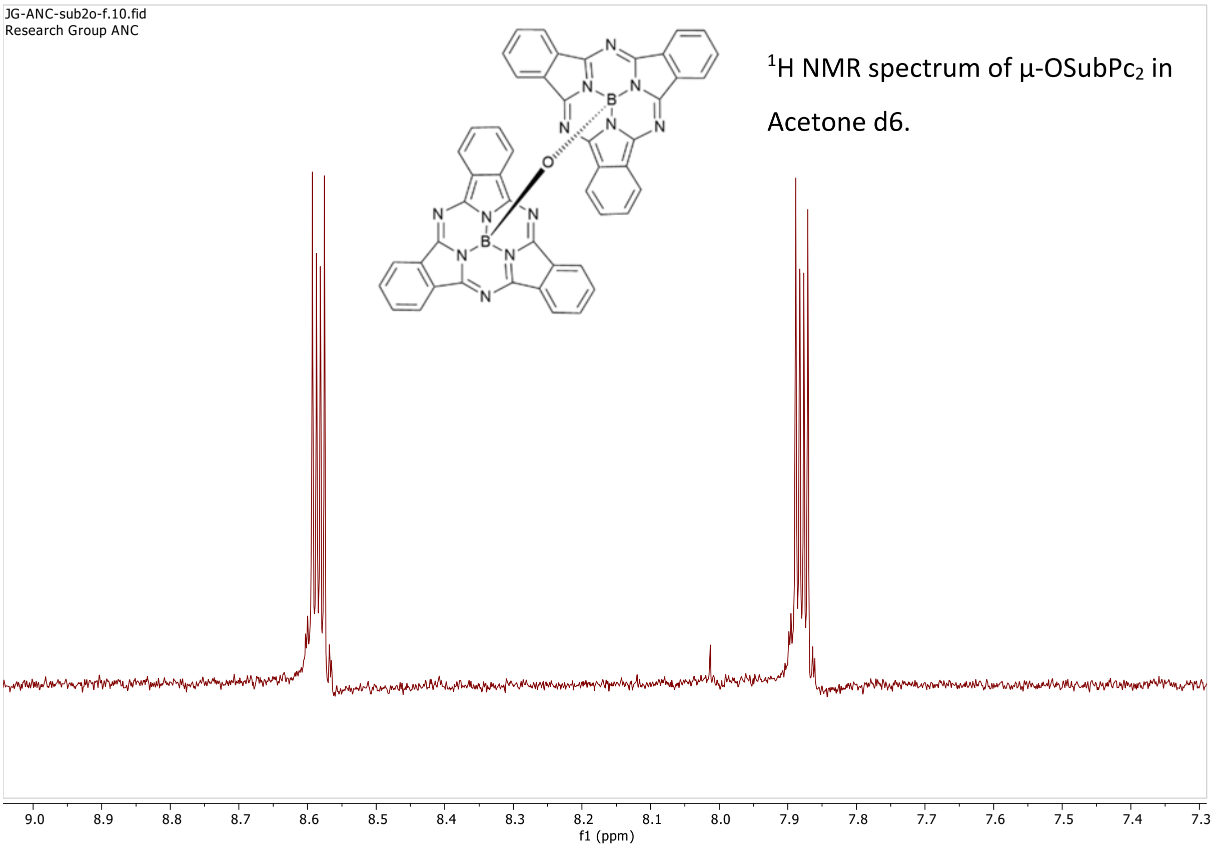

^{1}H NMR spectrum of μ-OSubPc$_2$ in Acetone d6.

References

[1] C. R. Hall, J. Conyard, I. A. Heisler, G. Jones, J. Frost, W. R. Browne, B. L. Feringa, S. R. Meech, *Journal of the American Chemical Society* **2017**, *139*, 7408-7414.

[2] J. J. Snellenburg, S. P. Laptenok, R. Seger, K. M. Mullen, I. H. M. van Stokkum, *Journal of Statistical Software* **2012**, *49*, 1-22.

[3] M. J. Frisch, G. W. Trucks, H. B. Schlegel, G. E. Scuseria, M. A. Robb, J. R. Cheeseman, G. Scalmani, V. Barone, G. A. Petersson, H. Nakatsuji, X. Li, M. Caricato, A. V. Marenich, J. Bloino, B. G. Janesko, R. Gomperts, B. Mennucci, H. P. Hratchian, J. V. Ortiz, A. F. Izmaylov, J. L. Sonnenberg, Williams, F. Ding, F. Lipparini, F. Egidi, J. Goings, B. Peng, A. Petrone, T. Henderson, D. Ranasinghe, V. G. Zakrzewski, J. Gao, N. Rega, G. Zheng, W. Liang, M. Hada, M. Ehara, K. Toyota, R. Fukuda, J. Hasegawa, M. Ishida, T. Nakajima, Y. Honda, O. Kitao, H. Nakai, T. Vreven, K. Throssell, J. A. Montgomery Jr., J. E. Peralta, F. Ogliaro, M. J. Bearpark, J. J. Heyd, E. N. Brothers, K. N. Kudin, V. N. Staroverov, T. A. Keith, R. Kobayashi, J. Normand, K. Raghavachari, A. P. Rendell, J. C. Burant, S. S. Iyengar, J. Tomasi, M. Cossi, J. M. Millam, M. Klene, C. Adamo, R. Cammi, J. W. Ochterski, R. L. Martin, K. Morokuma, O. Farkas, J. B. Foresman, D. J. Fox, Wallingford, CT, **2016**.

[4] J. D. Dang, M. V. Fulford, B. A. Kamino, A. S. Paton, T. P. Bender, *Dalton Transactions* **2015**, *44*, 4280-4288.

[5] D. B. Sulas, E. J. Rabe, C. W. Schlenker, *Journal of Physical Chemistry C* **2017**, *121*, 26667-26676.

[6] A. Weitemeyer, H. Kliesch, D. Woehrle, *The Journal of Organic Chemistry* **1995**, *60*, 4900-4904.

[7] M. Geyer, F. Plenzig, J. Rauschnabel, M. Hanack, B. del Rey, A. Sastre, T. Torres, *Synthesis* **1996**, *1996*, 1139-1151.
